# Online case-based learning in medical education: a scoping review

**DOI:** 10.1186/s12909-023-04520-w

**Published:** 2023-08-09

**Authors:** Rebecca Donkin, Heather Yule, Trina Fyfe

**Affiliations:** 1 School of Medicine and Dentistry, Griffith University, Sunshine Coast Health Institute, 6 Doherty St, Birtinya, Qld 4575 Australia; 2 School of Health, University of the Sunshine Coast, 90 Sippy Downs Drive, Sippy Downs, Qld, 4556 Australia; 3 Department of Cellular and Physiological Sciences, Faculty of Medicine, University of British Columbia, 2350 Health Sciences Mall, Vancouver, BC V6T 1Z3 Canada; 4 Division of Medical Sciences, University of British Columbia, 3333 University Way, Prince George, BC V2N 4Z9 Canada

**Keywords:** Case Based Learning, Medicine, Online delivery

## Abstract

**Background:**

Case-Based Learning (CBL) in medical education is a teaching approach that engages students as learners through active learning in small, collaborative groups to solve cases from clinical patients. Due to the challenges afforded by the COVID-19 pandemic, small group learning such as CBL, transitioned quickly to include technology-enhanced learning to enable distance delivery, with little information on how to apply pedagogical frameworks and use learning theories to design and deliver online content.

**Methods:**

To extend understanding of online CBL a scoping review protocol following the PRISMA-ScR framework explored the literature that describes the use of online CBL application in medical education and the outcomes, perceptions, and learning theories. A literature search was conducted in January 2022 followed by a subsequent review in October 2022. After peer review using the PRESS guidelines, the CASP appraisal tool was used to assess the rigor of each study design.

**Results:**

The scoping review identified literature published between 2010 and 2022 (*n* = 13 articles), on online CBL in the field of medical education with 11 observational studies describing student and facilitator perceptions and two randomized controlled studies. Positive perceptions of online learning included a flexible work-life balance, connection with learners, and improved accessibility. Negative experiences of online CBL included poor internet access, a distracting learning environment, and loss of communication. In the studies that collected student performance data, results showed equivalent or improved outcomes compared to the control. The CASP appraisal tool highlighted the deficiencies in most study designs, lack of framework or learning theory, and poor reproducibility of the methods to answer the research questions.

**Conclusion:**

This scoping review identified literature to describe the academic outcomes, and student and facilitator perceptions of online CBL in medical education. However, the CASP tool uncovered deficiencies in study descriptions and design leading to poor quality evidence in this area. The authors provide recommendations for frameworks and learning theories for the future implementation of online CBL.

**Supplementary Information:**

The online version contains supplementary material available at 10.1186/s12909-023-04520-w.

## Background

 Small group learning such as Case-Based Learning (CBL) is a teaching approach that engages students as learners through active learning in small, collaborative groups to solve problems that resemble real-world examples. [1] In medical education, the specific scenarios and problems would be based on contextualized cases from clinical patients [2]. Participants build their knowledge and work together as a group (from six to ten students) with a facilitator over one or more sessions. The format is versatile and can focus on one simple case scenario or a more complex case that requires multiple sessions with additional learning resources. The facilitator is ideally a content expert and corrects misconceptions or redirects students to the focused learning objectives [3]. This method is learner-centered and moves away from a didactic approach, with the interaction between students being the primary focus for inquiry [4]. In CBL, students are encouraged to develop skills in communication and critical thinking while receiving feedback on participation and preparation from their peers and facilitator to improve learning through a case-based approach.

Although there is a disparity in the definition of CBL with similar yet distinctly different teaching methods such as problem-based learning (PBL), [5] small group learning has been used in medical fields since 1912 [6] and is situated between structured and guided learning. [2] There is a wealth of research on small group learning for PBL and CBL, that has helped understand many aspects and elements of collaborative learning. Cen et al. [7] completed a meta-analysis in 2021 that described randomized control trials comparing CBL with other teaching methods in medical student education. Results from this study described how CBL teaching can improve medical students’ academic performance. However, the meta-analysis only reported eight articles, and none of these were compared to online CBL [7].

While CBL and PBL, small group learning pedagogies are not new, the trend of medical programs moving away from in-person delivery of learning has manifested over time to include a hybrid of technology-enabled learning (TEL) with in-class design [8, 9]. Online or TEL small group learning was introduced to harness the potential of asynchronous and synchronous collaborative learning that would give the student a platform for continuous interactions and engagement [10] and reduce the need for face-to-face interactions at the same place and time that limits the availability of expert staff, timetabling physical learning spaces and flexibility for the student [11].

Particularly, the delivery of small-group learning utilizing TEL has progressed in recent years mainly from the challenges afforded by the COVID-19 pandemic with a reduction in face-to-face classes and an inability to teach in a clinical setting which has rapidly driven online or remote delivery. Early evidence from undergraduate science courses has reported CBL delivered online as a comparable learning experience to in-class CBL delivery. [12] However, developing small group learning through a TEL lens and examining the student perception of learning has not been well researched in medical education but has been evaluated in other health (non-medical) programs. [9, 13, 14].

Whilst many institutions, including the author’s institutions, quickly adapted to online delivery throughout the pandemic using TEL to support online CBL such as WeChat, [15] eLearning, [16] and online platforms such as Zoom [17] there was little published information available on the rate of uptake in medical education and how to best deliver online small group learning that was previously conducted in-person. Furthermore, there is a paucity of literature that details the learning theories and outcomes of online CBL use in medical education.

To extend understanding in this area a scoping review was chosen as the purpose of the review was to identify knowledge gaps and scope a body of literature, to examine how research is conducted on a certain topic or field. [18] Scoping reviews are also useful for examining emerging evidence when it is still unclear what other, more specific questions can be posed and valuably addressed by a more precise systematic review [19]. The scoping review in this study explores the evidence-based literature that describes the use of online CBL application in medical education and how this literature describes CBL group work, outcomes, perceptions, and learning theories. Findings from this review will identify areas for improvement in online CBL and highlight a framework for the future direction that fits with curriculum design principles in a university setting and furthermore, if a full systematic review is warranted.

## Methods

### Preliminary search and protocol registration

A scoping review provides researchers the opportunity to look broadly at the literature with a focused question, identify gaps, and map existing literature [20]. Scoping reviews are beneficial for reviewing the literature on the breadth of the topic that is unclear or evolving and appropriate for questions that are meant to inform practice that has not been extensively and comprehensively examined [21, 22] , such as identified in this review. Both the PRISMA-ScR (Preferred Reporting Items for Systematic reviews and Meta-Analyses extension for Scoping Reviews) and the Joanna Briggs Institute 2020 guide [23, 24] were used to map and report results of the search and review process.

On the 28th of May 2021, a preliminary search was conducted to identify any existing reviews on the specified topic. The search was conducted by an experienced librarian (TF) using the PubMed and Ovid MEDLINE databases using keywords AND/OR Boolean operators determined from the literature and consultation with experts in the field. Examples of keywords included, “case-based learning OR CBL” AND “online OR virtual* OR web based* OR electronic* OR remote OR distance” AND “medical education OR pre-clinical OR undergraduate OR first-year* or second-year*” in titles, abstracts, and keywords of articles. PubMed identified 84 articles that were published on this topic, and Ovid MEDLINE identified 60 articles. There were no scoping reviews identified in this search. From this preliminary study, a full scoping review protocol was registered and published (10.25907/00071) outlining the methodology of the scoping review protocol including the search strategy and eligibility criteria [25]

### Identifying the research question

The objective of the scoping review was to identify and describe the student and facilitator perceptions of online CBL in medical education. This prompted three specific research questions.

1. What are the frameworks and learning theories of online CBL?
2. How has online CBL been applied in medical education?
3. What are the student and facilitator perceptions and student performance outcomes of online CBL in medical education?

### Identifying relevant studies

A full literature search was conducted by TF on January 31, 2022, followed by a subsequent review on October 14, 2022, to capture any new articles. To maintain rigor the search was peer-reviewed by an independent librarian following the guidelines from the Peer Review of Electronic Search Strategies (PRESS) for systematic reviews [26]. The Search results were imported into Covidence systematic review software (Veritas Health Innovation, Melbourne, Australia) which enabled all authors to review, extract data against eligibility criteria (Table 1), collaborate online from anywhere, and capture articles as per the PRISMA-ScR flow chart.

**Table 1:** Inclusion and exclusion eligibility criteria

Criterion	Inclusion	Exclusion
Study Design	Random control trials, observational cohort studies, quantitative and qualitative studies	Reviews, opinions, or commentaries
Population	Academic or student participants in pre-medical or undergraduate medical education in a university framework	Postgraduate or fellowship medical education not under a university guideline, non-medical undergraduate or postgraduate education e.g., undergraduate nursing
Type of Study	Investigation into the student and academic perceptions and grade outcomes in online CBL	Investigations in face-to-face/on campus CBL, or studies that are pre-clinical but do not relate to online CBL, or hybrid models of both face-to-face and online
Outcomes	Student and/or academic perceptions of online CBL and student grade or progression outcomes, along with learning theories and frameworks.	No student/academic perception and/or no student grade outcomes, inability to define learning theory/framework.
Language	Published in English	Published in languages other than English
Source	Original research papers, peer reviewed journal articles. (Review articles will be screened for broader context, further insights and references)	Conference abstracts, editorials, letters to the editor, newspaper articles, case reports and/or case series
Publication Dates	All	

### Selecting the studies to be included

To reduce potential selection bias, RD and HY independently reviewed the titles and abstracts of all articles. The resulting list of full-text articles were reviewed independently for inclusion in the final review. To facilitate calibration, frequent iterative online Zoom meetings were held during the process with the first meeting focused on creating a shared understanding of the criteria and then subsequent meetings comparing selected articles and discussing any discrepancies. Any unresolved discrepancies were resolved through a third reviewer (TF) with extensive experience in performing reviews who had final input in decision making, through discussion as an iterative process.

The Critical Appraisal Skills Programme (CASP) qualitative, cohort study, and randomized controlled study checklists were used to appraise quality depending on the study design [27]. Study quality is not a criterion for scoping reviews, however, all authors deemed it important to provide quality assessments of the included studies for the purpose of this review.

### Charting the data

A data extraction tool [25] was created that included and expanded upon the PRISMA-ScR checklist [24] and was subsequently piloted by independently reviewing 10 articles and then comparing results. The extraction tool was modified based on the pilot that all articles must contain an explicit definition or description of the method that described small group learning (two or more students) delivered as an online clinical case or scenario, not as an independent activity. This excluded independent eLearning modules or platforms that contained cases that were completed independently and did not require communication in small group learning.

### Collating, summarizing, and reporting results

Key findings of results, implications, and recommendations were tabled to examine the extent, range, and nature of the teaching intervention. Thematic analysis of positive and negative student and facilitator perception and student performance data of learning through online CBL was captured from the scoping review to summarise and disseminate research findings. The reporting of results was also conducted to determine the feasibility of undertaking a full systematic review.

### Undertaking consultation

The preliminary findings were shared with stakeholders from the Centre for Health Education Scholarship who were faculty members in medical education to understand if and in what ways the findings resonated with their experiences of conducting small group learning delivered online in medical education during the COVID-19 pandemic. The six stakeholders agreed with the findings from the literature and added additional comments regarding, the lack of learning frameworks, communication issues due to technology, and social issues in small group learning. This is further incorporated in the discussion along with key recommendations to improve online CBL.

## Results

**Fig. 1:**
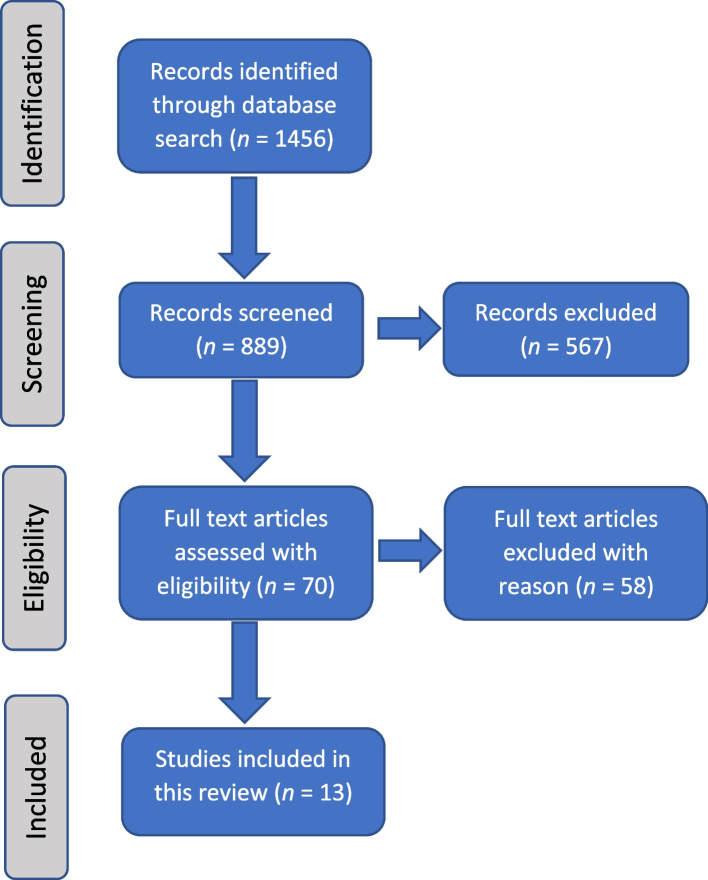
Study search strategy flowchart

There were 1456 publications imported for screening, and after 567 duplicates were removed, 889 studies were screened. After applying the inclusion and exclusion criteria, 70 full-text studies were included to be assessed for eligibility. Of these, 58 studies were excluded which resulted in 13 articles [28–40] in the scoping review. The main reasons why articles were excluded were for the following reasons: conference proceedings; wrong cohort (for example not medical education); wrong study design (not online small group learning) or a hybrid design of mixed online computer simulated patient or e-Learning case in a face-to-face learning environment (not online small group design). Details of the study search strategy are outlined in Fig. 1.

### Publication characteristics

All 13 articles included in the review were published between 2010 and 2022. Articles were published both pre and during the COVID-19 pandemic, although the majority (*n* = 9) of the articles reported outcomes and perceptions during the COVID-19 pandemic. The date and duration of each study varied between three months to two years, with publications originating from India, Canada, the USA, Austria, Denmark, Germany, the United Kingdom, and Somaliland.

The majority of the studies (*n* = 12) evaluated the students’ perceptions of online CBL and were descriptive in nature. Approximately half (*n* = 7) of the studies had a mixed-method approach, assessing both summative grade outcomes and student perception. One study assessed student perception and formative outcomes. Approximately a third (*n* = 4) included a control or comparison group. Only three studies identified a learning theory or framework to guide their study design which was, the effectiveness of learning using different materials aligned with Bloom’s taxonomy (*n* = 1), and Kirkpatrick’s learning theory (*n* = 2).

The 13 studies reviewed included 3,540 student participants and two studies included a further 148 facilitators, with a total of 3,688 participants. All participants were medical students or facilitators teaching medical education. Supplementary file 1 details the publications as a final composite of tabulated data including the charted items as described in the protocol.

### Application of online case-based learning

The application of online CBL was diverse and included a variety of platforms to achieve online communication and resources. Communication ranged from online supported video-based group discussions (e.g., Zoom), or through platforms such as Google Classroom stream, blogs, or instant messaging. When described, online learning resources were shared through the university learning management system, publicly available online resources, websites, and platforms that enabled share sites supported by group learning. Online small group learning ranged from groups of 2–16 students or was simply described as “*small groups*”.

### Student and facilitator perceptions and outcomes of online case-based learning

The most common positive perceptions of online CBL included accessibility to advanced multimedia content with interactive activities along with developing analytical and critical thinking to benefit clinical knowledge. Other positive perceptions included a better learning environment, improved facilitation, promotion of technology-enhanced collaboration, and peer discussion through connection to groups at different locations. The predominant negative aspects were technology issues, which included network accessibility which reduced the ability to contribute to the discussion. Mukhopadhyay et al. [37] reported “*a sizable number of students (42.8%) experienced technology issues*” which was mostly attributed to poor internet connectivity and limited internet data. Communication issues occurred for both students and facilitators with the lack of visual guides and the inability to read body language online. A distracting learning environment (learning from home, social media, Zoom fatigue) was also perceived as a negative outcome of online CBL. When asked to compare the experience of online CBL with face-to-face CBL in the study by Dawson et al. [35] “*49% of students and 60% of facilitators rated the online CBL learning experience as much worse compared to in-person CBL*”. Comprehensive details of positive and negative perceptions of online CBL extracted from the scoping review are shown in Table 2.

**Table 2:**
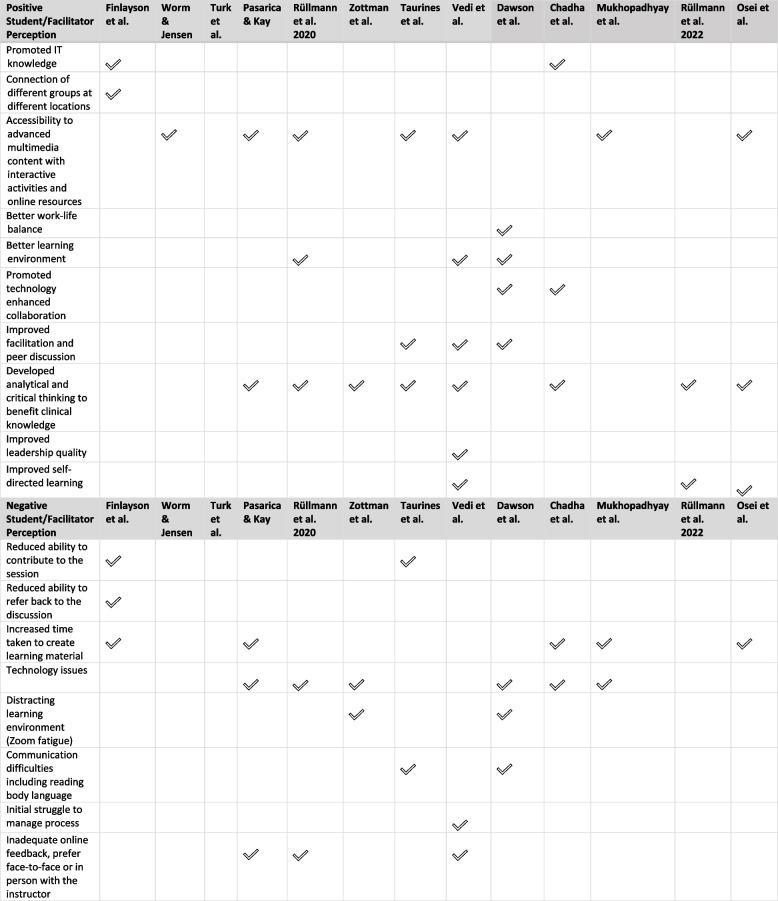
Positive and negative student/facilitator perceptions of online case based learning

For those studies that collected student performance and outcome data (*n* = 8 studies), there was a mostly positive response. Vedi et al. [34] reported a statistically significant learning result for online CBL demonstrated by pre and post MCQ after every case (37.2 vs. 41.78, *p* < 0.0416). Turk et al. [29] reported a learning benefit from online CBL that occurred in three steps (written resources provide base knowledge, practice application with online CBL, and then contact with real cases) with students achieving significantly higher objective structured clinical examination (OSCE) scores when comparing pre and post online CBL intervention (1.02 adjusted *p* = 0.002). Worm and Jensen [40] reported that online resources and the effect of online peer learning in groups increased learning satisfaction and improved knowledge demonstrated by improved pre-test and post-test scores (66% vs. 70%). Mukhopadhyay et al. [37] reported an increase in formative assessment when evaluating pre-test and post-test scores that were statistically significant (73.3% vs. 77.03%, *p* = 0.03). Other studies had mixed results when assessing competency based and diagnostic skills that had been taught online through small groups.

Chadha et al. [36] reported statistically significant improvement in overall pre and post-tests scores with knowledge gains from 57 to 70%. However, when subanalysis was conducted by individual topics, only three of the five topics had significant improvement in responses to knowledge questions post-test.

Rüllmann et al. [38] reported students had significantly improved descriptions of heart murmurs by virtual auscultation but there was no significant difference between the groups in diagnostic accuracy. The limitations discussed in this study included no assessment of transfer to in-person patient skills, or long-term retention so it was difficult to interpret if these results were favorable or not. The CASP appraisal tool assisted in further dissecting these findings along with assessing the rigor of all studies and the implications of their results.

### CASP appraisal tool for a cohort and randomized control studies

While there were reported positive student perceptions and improved student outcome data there were many limitations to the studies which included a small sample size, a single institution study, lack of study design reproducibility, or absence of a control group. Study quality is not a criterion for scoping reviews however, after reviewing the limitations of the articles it was deemed important to provide quality assessments using the Critical Appraisal Skills Programme (CASP) qualitative, cohort study, and randomized controlled study checklists. [27] The CASP appraisal tool was a useful item to capture answers to specific appraisal questions enabling objective insight through a traffic light system (Yes = green; Can’t tell = orange; No = red) into the rigor of the study design and the reliability and precision of the results in the study. Table 3 highlights the appraisal for the 11 cohort studies and the two randomized controlled studies. All studies focused on an issue relating to online CBL however, due to limited information or poor study design it was difficult to identify the precision of the results and whether there are implications of the study for practice or if the intervention (online) provided greater value than an existing intervention (face-to-face). Studies that provided clear answers to the appraisal questions are further deliberated in the framework for online CBL outlined in the Discussion.

**Table 3:**
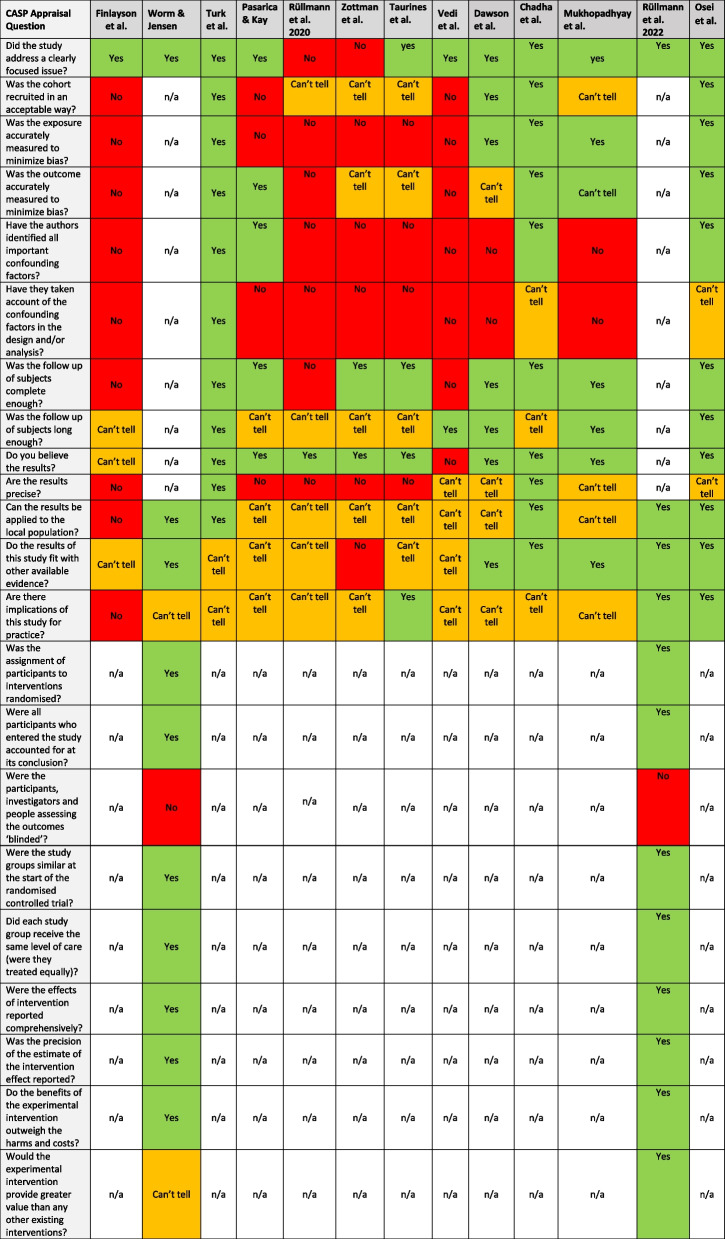
CASP appraisal tool to enable objective insight of studies through a traffic light system (Yes = green; Can’t tell = orange; No = red)

## Discussion

This scoping review has identified that there is a gap in the literature describing outcomes with online CBL use in medical education. This is mainly due to poor study designs without frameworks or learning theories to support evidence, limiting the feasibility of a full systematic review at this time. There is a lack of consistency in the description of key study components (population, intervention, and outcome measures), most likely due to studies describing a pilot or pivot response to online education due to the COVID-19 pandemic.

Of the 13 included studies, three were small, pilot studies conducted before the COVID-19 pandemic, and ten were medical program pivots due to COVID-19 restrictions with different populations and program years. The online intervention varied with diverse definitions of what constitutes online group work, whether it was CBL or another framework, and the length of the sessions. Multiple online platforms were used to support online small group learning including text messaging in earlier studies, and Zoom video conferencing, google groups, and WeChat in recent studies. There was also a variety of study designs from observational studies to randomized controlled trials. Outcome measures of student performance were determined by several different assessments including multiple choice tests, knowledge scores before and after the intervention, and OSCE skill tests. Whereas, student and facilitator perception measures were determined by surveys, interviews, and focus groups. Critical appraisal highlighted the lack of high-quality, reproducible evidence in this field and the limited learning theories and frameworks. The CASP appraisal tool enabled objective insight into studies both pre- and during COVID-19 pandemic (Table 3). The CASP appraisal tool easily identifies areas of strengths and weakness in all aspects of a study and there were no consistent advantages or disadvantages when comparing studies before or during the pandemic i.e., there were poor study designs without frameworks or learning theories both pre- and during the COVID-19 pandemic. However, as would be expected study designs that included a randomized controlled trial provided greater objective insight, and these study designs were included both pre- and during the COVID-19 pandemic.

### Small group learning frameworks and applications

The nuances between CBL and PBL, have been explored through their definitions and applications across medical education and other disciplines and have been used interchangeably for small group learning. Both originate from constructivist conceptions of learning by assuming that knowledge is constructed by the learners in their interactions with the environment [9, 41, 42]. A learner-centered design is the focus with an expert as a facilitator of learning, rather than the content expert [43] with students searching, evaluating, constructing and sharing information, to apply it in the context of the problem-solving process at hand [44]. Furthermore, these small group collaborative learning sessions have been influenced by sociocultural theory, which emphasizes the pursuit of common goals through interpersonal interactions [45, 46]. Albanese and Mitchell [47] describe it as a method of instruction as a basis for developing problem-solving skills using clinical patient problems as a catalyst to develop knowledge in the clinical sciences. In this setting the group learning process provides both structure and a social setting to problem solve through reflection in a systematic way [48].

The few studies included in this scoping review that investigated explicit learning outcomes (e.g., grades/scores) concluded that online CBL is either equivalent to or superior to traditional face-to-face delivery. However, the perceptions and implicit experience (e.g., social aspects, communication) of online CBL for participants were inferior in many studies. The social constructs that develop in small group collaborative learning can enhance perceived learning [4, 12] which can be linked to improved motivation and engagement inciting the practice of social constructivism and self-determination theory [49]. Both encapsulate the concepts of intrinsic and extrinsic motivation into autonomous (self-determined) and controlled motivation. [50] It follows that how students engage with online learning and their motivating factors need to be carefully considered because the socio-cultural learning environment can influence student satisfaction and the affective experience of learning online. [51].

The application of CBL in an online environment is similar however, the delivery is different. Subsequently, the learning theories and frameworks pertained to face-to-face learning may not be solely applicable to an online environment. However, it was difficult to establish if and how a learning theory or framework could be determined as successful in online small group learning as only three (21%) of the studies in this review applied a framework. One study applied Bloom’s taxonomy [52] to define and distinguish different levels of human cognition [40] and two studies [30, 39] used Kirkpatrick’s model [53] for training evaluation, neither is exclusive to online learning.

A recommendation when developing a study design of a new online course or a pivot curriculum is to include a framework that can scaffold the learning design and also assess the outcome. Frameworks used in business include the Indicators of Engaged Learning Online (IELO) framework first developed by Means [54] and subsequently updated by Bigatel and Edel-Malizia [55]. More recently a framework proposal by de Nooijer, [56] provides recommendations for optimizing collaborative learning in PBL using a constructive approach with an online lens that could be extended to include online CBL.

### Learning outcomes of online CBL

Previous literature that describes the results of online small group learning in health programs includes a variety of equivalent, positive and negative findings when compared to face-to-face cohorts. Examples include the following by Nicklen et al. [9] and Ng et al. [11] who reported a comparable learning experience to traditional CBL in physiotherapy students and PBL in speech/language pathology students. However, student dissatisfaction and decreased perceived depth of learning were reported in the remote CBL learning group [9] whereas students reported enjoying online PBL and decreased travel time to and from school. [11] Erickson et al. [14] also found similar results with students preferring more flexibility and accessibility as an alternative to face-to-face PBL when using focus group and survey data to evaluate PBL health science online. However, negative findings from this study included poor internet connectivity and reported difficulties with rapport building, and limited depth of discussion online. Facilitators in this study reported additional effort and preparation were required for online PBL compared to face-to-face PBL. Conversely, Leavy et al. [13] reported final marks were significantly higher for fully online health science students compared with face-to-face students and describe action research [57] as the learning theory for their study design. However, student perception of online learning and the quality of teaching were lower than the face-to-face group.

These findings in other health programs are not too dissimilar to those found in this online CBL scoping review for medical education. Information obtained from all of these studies can inform best practices in online small group learning education.

### Informing best practice

To overcome student or facilitator perceived shortcomings of online CBL (increased time spent on online learning, digital competency, and emotional demand), which leads to increased extraneous load through superfluous processes [58, 59] metacognitive support is required for both facilitators and students. Examples include the purposeful design of cases with clear learning and outcome objectives, recordings with accessibility captions, interactivity of online resources to promote engagement, timely and constructive feedback, facilitator flexibility, adaptability in an online environment, emotional support for digital literacy, and above all facilitators and students who are resilient, motivated and have a positive attitude to learn online [60]. Although most studies report learning outcomes are equivalent between face-to-face and online delivery, facilitator and student perceptions of online learning will change depending on the circumstances. This was evident throughout the COVID-19 pandemic whereby an immediate pivot to fully online courses occurred to enable learning to continue and for educators to remain teaching. However, the sustainability and perceptions to continue in this format have waned with the return to face-to-face education, fewer restrictions, and the ability for flexibility allowing a hybrid approach of both online and face-to-face learning. To further inform best practices and lessons learned from this review, five key recommendations for future planning and implementation of online CBL are summarized in Table 4.

**Table 4:** Lessons learned and recommendations for future planning and implementation of online Case Based Learning

*Lesson*	*Recommendation*
**1.** Current online CBL literature does not always describe the participant population, the intervention, or the outcomes measured. Additionally, study designs used to assess the effectiveness of online CBL programs lack rigor.	Higher quality evidence with clear descriptions of online CBL program components is needed in order to help plan future programs. CASP critical appraisal tools can be used to design and evaluate studies.
**2.** The majority of current online CBL programs are not planned using learning theories or frameworks.	A constructivist, learner-centered, social learning approach should be integrated into the online experience. Additionally, cognitive load and metacognitive support should be adjusted considering time requirements and fatigue experienced by online learners and facilitators.
**3.** Technology matters! Access to equipment, a fast reliable network, an education platform, and technical support are critical for online CBL program success.	Learners and facilitators need to be consulted and provided with equipment and training that addresses barriers related to technology issues. Additionally, highlighting the use of online teaching and learning skills beyond CBL such as telemedicine, virtual meetings, and remote continuing professional development is beneficial.
**4.** Medical programs are comprised of different courses where students learn both hard (knowledge, content, clinical skills) and soft (professionalism, communication, collaboration, leadership, advocacy) skills. Some courses appear better suited to the online learning environment than others.	High quality evidence is needed to help medical program directors develop a balanced curriculum with both in-person and virtual components. Specific courses may benefit from online delivery with improved access to remote experts and schedule flexibility.
**5.** The opportunity to engage with technology is often limited to high-income countries that have financial and infrastructure support. Some technology is not available or is inaccessible to resource-strained countries [61]. This disparity between high and low-income countries impacts the effectiveness of an online curriculum and the educational outcomes.	The COVID-19 pandemic has ignited the development of online education platforms and uncovered inequities in access to education. Let us move beyond the COVID-19 pandemic and share online medical education resources to bring global communities together to develop a culture of inclusivity.

### Limitations and future directions

As there was limited literature detailing online CBL in medical education the results should be interpreted with caution as this may not be consistent with all findings, this is particularly relevant to low income countries that may not have the necessary technology and high speed internet to meet the needs of online learning [61]. There are limitations to the application of learning theories that pertain to online small group learning as this has not been fully evaluated in medical education and would likely differ between programs, institutions, and training provided for both staff and students. This scoping review could not evaluate long-term outcomes beyond three months and whether transferrable skills are impacted by online delivery. It would be of interest to conduct further studies evaluating long-term outcomes in residency training and whether online CBL, particularly held during the COVID-19 pandemic, influenced future learning. Perceptions and outcomes of online CBL should not be limited to student and facilitator’s experience but also at the organizational or management level with perceived or actual financial and curriculum detriments or benefits but, this was beyond the scope of the study.

## Conclusion

There is limited literature on online CBL for medical education. We expect that post COVID-19 pandemic there will be an explosion of research in this area as institutions navigate a new curriculum that encompasses a hybrid approach of necessary in person clinical skills education that is supplemented with online learning. There are many benefits of including online education in a curriculum (including medical education) however, learning theories and frameworks should be considered in the design such as a constructivist, learner-centered, social learning approach. From this scoping review and literature grounded in evidence from other disciplines, key recommendations have been suggested to assist in designing an online CBL curriculum. Despite its limitations, this scoping review followed an appropriate method using a five-stage framework that was independently verified. The inclusion of the CASP appraisal tool afforded an opportunity to easily assess the rigor of each study and demonstrated the need for recommendations to design a quality curriculum.

In conclusion, it is anticipated this article will stimulate discussion and proactive design to support online learning, particularly in medical education. A review of the lessons learned pre and post the COVID-19 pandemic will improve online medical education and establish practices that enhance small group learning.

### Supplementary Information

**Additional file 1: Supplementary file 1. **Summary of included studies.

## Data Availability

The datasets used and/or analysed during the current study are available from the corresponding author on reasonable request.
